# Gastric Inhibitory Polypeptide (GIP) Is Selectively Decreased in the Roux-Limb of Dietary Obese Mice after RYGB Surgery

**DOI:** 10.1371/journal.pone.0134728

**Published:** 2015-08-12

**Authors:** Jiaqiang Zhou, Zheng Hao, Nigel Irwin, Hans-Rudolf Berthoud, Jianping Ye

**Affiliations:** 1 Department of Endocrinology, Sir Run Run Shaw Hospital, Zhejiang University School of Medicine, Hangzhou, Zhejiang, China; 2 Antioxidant and Gene Regulation Laboratory, Pennington Biomedical Research Center, Louisiana State University System, Baton Rouge, Louisiana, United States of America; 3 School of Biomedical Sciences, University of Ulster, Coleraine, United Kingdom; 4 Neurobiology of Nutrition Laboratory, Pennington Biomedical Research Center, Louisiana State University System, Baton Rouge, Louisiana, United States of America; College of Tropical Agriculture and Human Resources, University of Hawaii, UNITED STATES

## Abstract

Gastric inhibitory polypeptide (GIP, glucose-dependent insulinotropic polypeptide) is expressed by intestinal K cells to regulate glucose-induced insulin secretion. The impact of Roux-en Y bypass (RYGB) surgery on blood GIP is highly contraversial. This study was conducted to address the mechanism of controversy. GIP mRNA was examined in the intestine, and serum GIP was determined using Luminex and ELISA in diet-induced obese (DIO) mice. The assays were conducted in RYGB mice in fasting and fed conditions. Food preference, weight loss and insulin sensitivity were monitored in RYGB mice. In DIO mice, GIP mRNA was increased by 80% in all sections of the small intestine over the lean control. The increase was observed in both fasting and fed conditions. After RYGB surgery, the food-induced GIP expression was selectively reduced in the Roux-limb, but not in the biliopancreatic and common limbs of intestine in fed condition. Lack of stimulation by glucose or cholesterol contributed to the reduction. Jejunal mucosa of Roux-limb exhibited hypertrophy, but villous surface was decreased by the undigested food. Serum GIP (total) was significantly higher in the fasting condition, but not in the fed condition due to attenuated GIP response to food intake in RYGB mice. The GIP alteration was associated with chow diet preference, sustained weight loss and insulin sensitization in RYGB mice. RYGB increased serum GIP in the fasting, but not in the fed conditions. The loss of food-induced GIP response in Roux-limb of intestine likely contributes to the attenuated serum GIP response to feeding.

## Introduction

Roux-en-Y gastric bypass surgery (RYGB) is a powerful therapy for obesity and type 2 diabetes [1, 2]. The surgery induces a negative energy balance by reducing food intake [2, 3] and increasing energy expenditure [4–7]. These changes are required for prevention of weight regain and diabetes recurrence. In addition to the anatomical alterations, changes in gut hormone secretion have been suggested for the negative energy balance. These hormones include glucagon-like peptide 1 (GLP-1) [8–10], peptide YY (PYY), GIP (gastric inhibitory polypeptide or glucose-dependent insulinotropic polypeptide), cholecystokinin (CCK), ghrelin and amylin [11, 12]. Studies from this and other groups suggest that PYY [13] and leptin [14], but not GLP-1 [15, 16], may contribute to the metabolic effects of RYGB. However, the role of GIP remains to be determined.

GIP is secreted by K cells of the epithelial lining in small intestine. The secretion is induced by food intake. Bioactive GIP is generated from precursor through cleavage of single arginine residue from the precursor by the intestine-specific prohormone convertase. GIP promotes energy storage by its action in multiple organs including pancreas, gut, adipose tissue and brain. GIP stimulates insulin secretion in β-cells, inhibits gastric acid secretion, suppress lipolysis in adipose tissue, induce appetite and reduce energy expenditure in the brain [17]. Inhibition of GIP activity by GIP-receptor gene knockout partially prevented diet-induced obesity in mice [18], suggesting that a decrease in GIP activity may lead to weight loss. Although this possibility has been tested in weight loss by RYGB surgery, there is controversy regarding serum GIP levels in RYGB subjects. Serum GIP was reported to be increased [19, 20], decreased [21–23] or unaltered [24–26] in RYGB patients. In RYGB rats, postprandial serum GIP was not changed in a study of 20–90 minute feeding [27], but was increased in a study of 15 minute feeding [28]. There is no report about GIP expression in the intestine after RYGB surgery. Lack of information about GIP mRNA expression in RYGB models may contribute to the discrepancy about plasma GIP. We addressed this issue by testing gut GIP mRNA in our mouse RYGB model.

In this study, GIP mRNA was examined in all segments of intestine in DIO mice before and after RYGB surgery. The results suggest that intestinal GIP mRNA was increased by high fat diet (HFD) and reduced in the Roux-limb by RYGB surgery. The reduction accounts for decreased food response of serum GIP in RYGB mice.

## Materials and Methods

### Diet-induced obese (DIO) mice and RYGB surgery

This study was conducted in mice and all procedures were approved by IACUC of the Pennington Biomedical Research Center, Louisiana State University, Baton Rouge, LA, USA. Six week-old C57BL/6J mice were purchased from the Jackson Laboratory or obtained through in-house breeding. The mice were fed a high fat diet (HFD, D12331 diet, 58% calories from fat, Research Diets Inc.) for 14 weeks to generate the diet-induced obese (DIO) model. RYGB surgery was performed in the mice at body weight around 50 g. The surgical operation for RYGB and sham was conducted as described previously [5]. In the sham operation, the perigastric ligaments were cut, and then a 3 mm incision was made in the stomach wall and closed with a titanium clip. In the cohorts 1–3, after surgery, the mice were fed medium-fat breeder chow diet (25% calorie from fat, 5015 LabDiet) to mimic dietary changes after RYGB surgery in patients [29, 30]. There are four cohorts in this study. In the first cohort, chow-fed and HFD-fed mice were compared for intestinal GIP mRNA in both overnight fasting and non-fasting conditions (freely fed) (n = 5). In the second cohort, GIP mRNA was examined in the intestine at 6 weeks after RYGB surgery (n = 5), in which mice were fed HFD before surgery and breeder chow diet after surgery. In the third cohort (n = 7–8, 8 shames and 7 RYGB), insulin sensitivity and serum gut hormones were determined 6–8 weeks after RYGB with the same dietary regimen. Weight-matched mice were generated by calorie-restriction of DIO mice on the breeder chow diet to induce weight reduction to match weight loss in RYGB mice. In the fourth cohort (n = 4), food intake was examined in animals with a choice between HFD and breeder chow diet after surgery. Food intake was measured daily during the first 6 weeks after surgery. The data were derived from 4 cohorts of studies to obtain data in different conditions. The mice were sacrificed by cervical dislocation for sample collection at the end of each study.

### Body weight, composition and insulin tolerance test

These parameters were measured using protocols as previously described [5]. Body weight was measured in mice weekly in the first 5 weeks and biweekly thereafter after surgery. Body composition was determined using NMR at 7 weeks after surgery. ITT was conducted in mice at 8 wks after RYGB. DIO mice were used in surgery at body weight of 50±3 g after 14 wks on HFD. In terms of adiposity, fat content was above 30% in DIO mice at this body weight.

### GIP mRNA and protein

In cohort 1 and 2, the intestine sample was collected immediately after mouse sacrifice, quickly cleaned, frozen in liquid nitrogen and then stored in -80°C. Collection was done in both overnight fasted mice and freely fed mice. Total RNA was prepared from the tissues with TRIzol reagent (Cat. T9424, Sigma, St. Louis, MO). TaqMan RT-PCR reaction was used to quantify GIP mRNA using the 7900 HT Fast real-time PCR System (Applied Biosystems, Foster City, CA) as previously described [31]. TaqMan primer for mouse GIP (Mm00433601-m1) and 18S (AIQJA2B, P N4331348) were purchased from the Applied Biosystems. The GIP mRNA signal was normalized with ribosome 18S RNA. In the tissue culture, GIP protein in the culture supernatant was determined using an ELISA kit (EZRMGIP-55K, EMD Millipore, Billerica, MA 01821). In cohort 1, GIP mRNA was tested at 10 wks on HFD. In cohort 2, the test was done at 6 weeks after surgery.

### Tissue culture

The small intestine (jejunum) was collected from lean mice at 7 wks of age in the fasting condition,and washed with cold DMEM to remove the intestinal content. The tissue was cut into small pieces around 1 mm in diameter, as previously described [32]. The tissue was treated for 2 hrs with nutrients such as glucose (Glu, 20 mM), cholesterol (CHO, 5 mM), linoleic acid (300 μM), palmitic acid (100 μM), and BSA (3%) in a 24 well-plate. Tissues were then collected after the treatment and used for GIP mRNA and protein assay.

### Serum GIP, insulin, PYY and ghrelin

Gut hormones were measured in mouse serum at 6 wks post-surgery in cohort 3 and 4. In cohort 3, blood samples were collected from the retro-orbital plexus in overnight fasted or at 30 min meal after overnight fast (postprandial condition). DPP4 inhibitor (Sitagliptin) was used to preserve incretines. The hormones were determined using the Luminex technology with a Mouse Serum Adipokine multiplex Kit (Cat. #MMHMAG-44K, EMD Millipore Corporation, 28820 Single Oak Drive, Temecula, California) according to the manufacturer's instruction. The assay provides information for total GIP protein. In cohort 4, serum GIP was examined 15 mins after glucose gavage (2 g/kg) using a rat/mouse GIP ELISA kit (total GIP, Cat. # EZRMGIP-55K, EMD Millipore Corporation). GIP protein concentration was determined according to the standard curve in the multiplex kit. Total ghrelin was determined in this assay.

### Hematoxylin and eosin (H&E) staining

Intestine tissues was collected immediately after mouse sacrifice, quickly cleaned, and fixed in 10% neutral buffered formalin solution (HT50-1-2; Sigma). The tissue slides were obtained through serial cross-section cutting at 8 μm thickness and processed with a standard HE staining procedure.

### Insulin tolerance test

In cohort 3, insulin tolerance test was conducted 8 wks after surgery with peritoneal injection of insulin (0.8 U/kg). Glucose was measured at 0, 30, 60 and 90 mins with a glucometer (Free style OneTouch, TheraSense, Phoenix, AZ) after the injection.

### Statistical Analysis

In the statistical analysis, two-tailed, unpaired Student’s t test was used in analysis of in vitro data, and one-way ANOVA was used in analysis of in vivo data with significance P <0.05.

## Results

### GIP mRNA was increased in the gut of DIO mice

**Fig 1:**
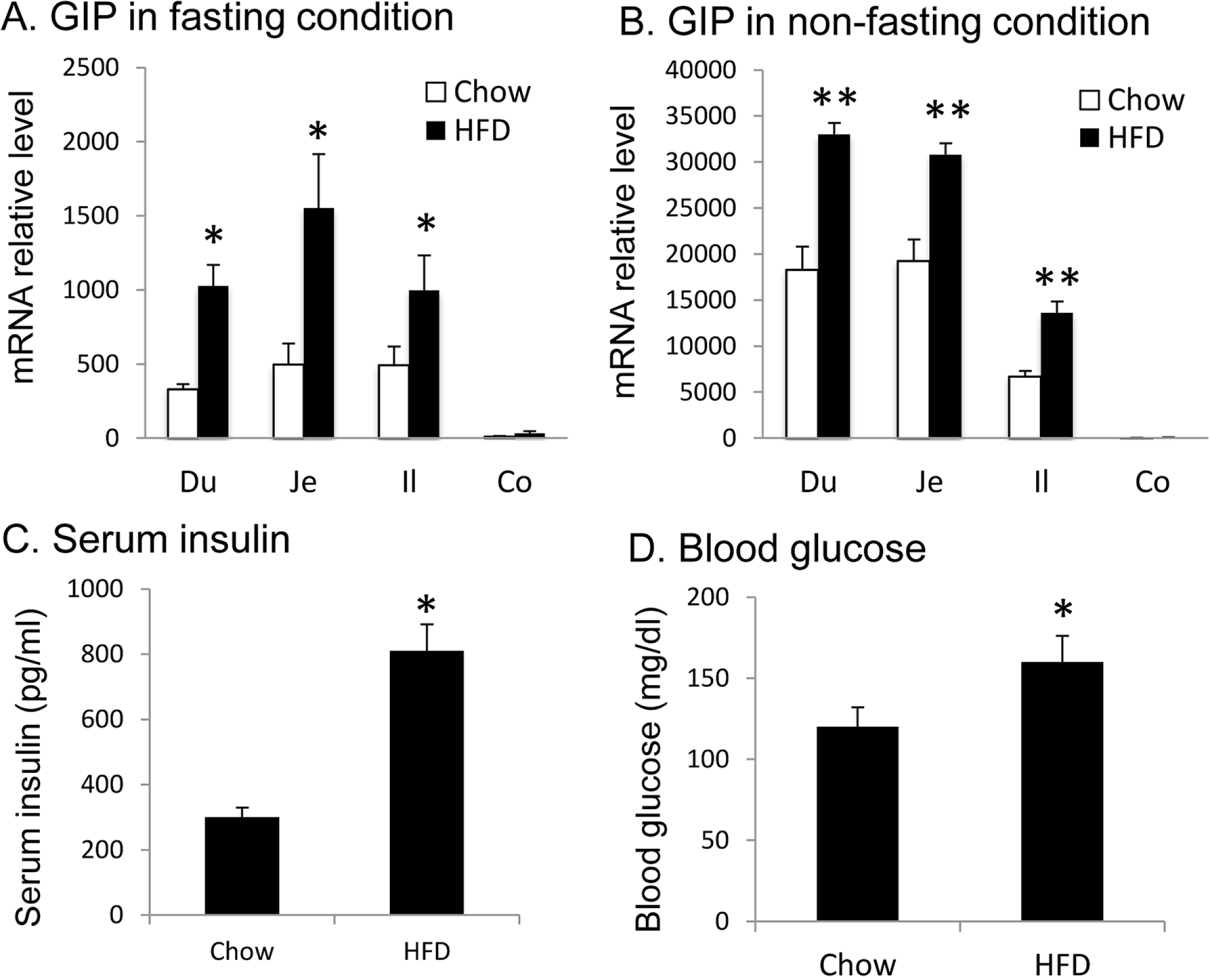
Association of intestine GIP mRNA and fasting insulin or glucose in DIO mice (Cohort 1). GIP expression was determined in DIO mice at 10 wks on HFD. A. GIP mRNA in the fasting condition. The expression was examined in duodenum (Du), jejunum (Je), ileum (Il) and colon (Co) in mice after overnight fast. Chow-fed mice were used as lean mice in the control. B. GIP mRNA in fed mice. Samples were collected in the morning after mouse food intake at night. C. Fasting insulin. The test was done after overnight fast. D. Fasting glucose. Data is presented as mean ± SE (n = 5). * p<0.01; ** p<0.001 RYGB vs. lean by student’s t test.

GIP mRNA expression was determined in the small and large intestine of lean and DIO mice in both the fasting and freely fed state. In chow-fed lean mice, mRNA was mainly detected in the small intestine including duodenum (Du), jejunum (Je), and ileum (Il) in the fasting condition (Fig 1A). The expression was a hundred fold less in colon (Co) (Fig 1A). GIP expression was increased in the freely fed state by over 30-fold in the duodenum, jejunum, and 10-fold in the ileum (Fig 1B). GIP expression was also increased in colon in the fed state, but the change was small compared to those in other regions of the small intestine (Fig 1B). In DIO mice, GIP expression in the small and large intestine was more than double that of lean mice in the fasting state (Fig 1A). In the freely fed state, GIP expression remained higher in the DIO mice, with at least 80% increase over lean mice (Fig 1B). Fasting insulin and blood glucose were significantly elevated in DIO mice (Fig 1C and 1D). Taken together, intestinal GIP expression is significantly induced in DIO mice regardless of feeding condition. The increase is associated with hyperinsulinemia and hyperglycemia.

### RYGB results in weight and fat loss

**Fig 2:**
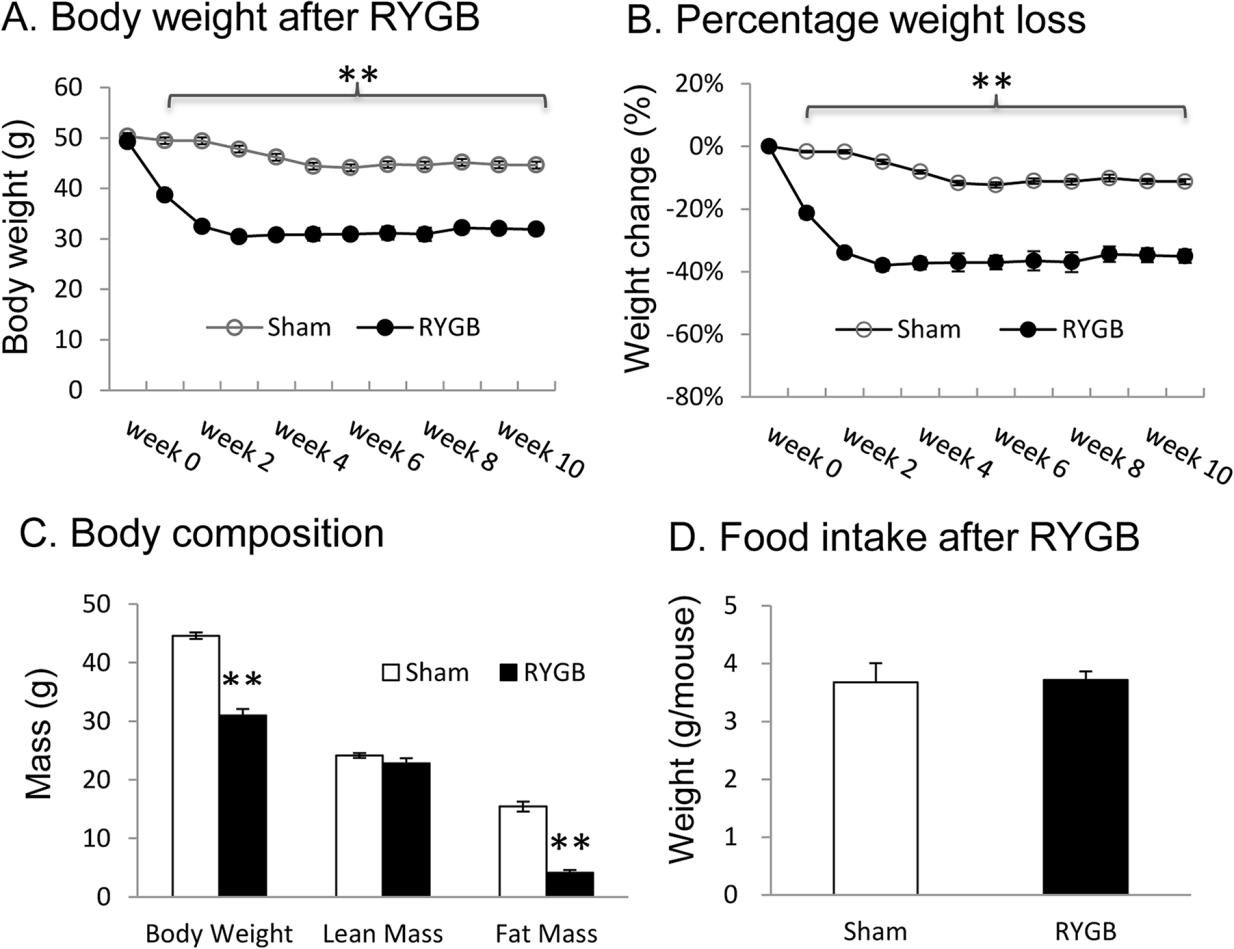
Weight loss in RYGB mice (Cohort 3). RYGB surgery was performed in DIO mice at 14 wks on HFD with body weight around 50g, and sham operation was performed in the control mice. After surgery, the mice were fed chow diet (11% fat wt/wt, 5015 LabDiet) to mimic the dietary condition in patients. A. Body weight change. B. Percentage change in body weight. C. Body composition. The composition test was conducted at 7 wks after surgery. D. Food intake at 6 wks after surgery. The data are presented as mean ± SE (n = 7–8). * P<0.01, ** p<0.001 RYGB vs. sham by student’s t test.

The RYGB mouse model was established using a protocol as previously described [5]. RYGB drastically reduced body weight by about 30%, which was entirely from fat loss (Fig 2, A-C). Lean body mass was not reduced by RYGB (Fig 2C). Maximal weight loss was achieved 2 wks after surgery and body weight was maintained at this lower level thereafter in the 12 wks study. Food intake was measured 6 wks post-surgery, and no difference was observed between RYGB and sham mice (Fig 2D).

### RYGB induces a transient reduction in food intake

**Fig 3:**
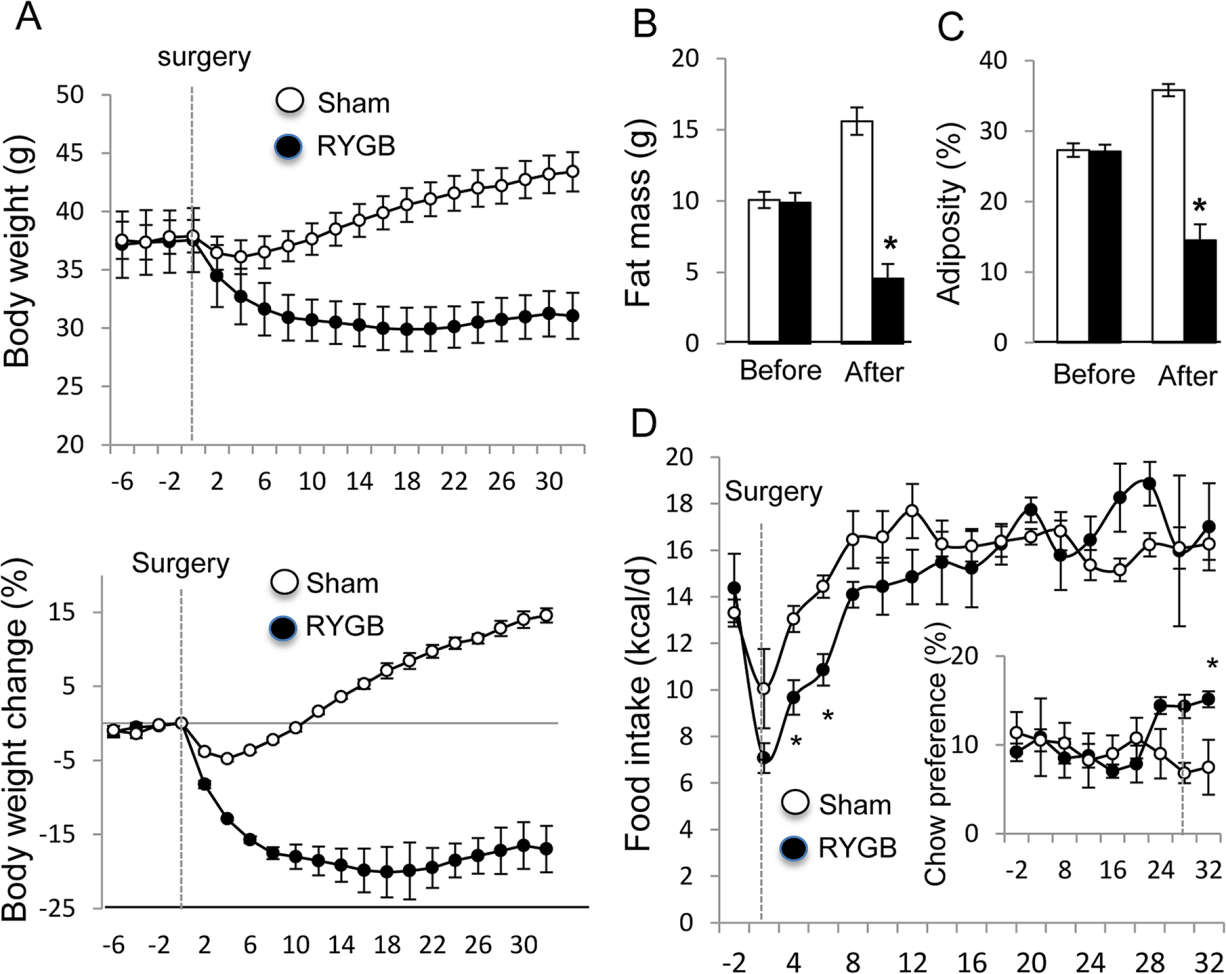
RYGB effects on body weight, body composition, food intake and food choice (Cohort 4). Mice were on HFD for 12 weeks before surgery and then on breeder chow and HFD for choice after surgery. A. Body weight change in gram and percentage after surgery. The percent body weight change was calculated from baseline before surgery. B. Fat mass reduction after surgery. C. Adiposity index before and after surgery at 35 days. D. Food intake and diet preference. Calorie intake was calculated in each mouse for combined HFD and breeder chow intake. Relative preference for breeder chow vs. HFD is presented (inset). The data are presented as mean ± SE (n = 4). * p< 0.05 RYGB vs. sham.

In cohort 3, sham-operated mice lost about 8% of body weight and no food reduction was observed in RYGB mice at 6 wks post-surgery. The weight loss may be a result of change from HFD to breeder chow diet. Food reduction may happen before 6 wks post-surgery. These possibilities were tested in cohort 4, in which mice were provided with HFD and breeder chow diet at the same time post-surgery, and food intake was monitored daily before and after surgery. The food choice was provided since RYGB mice do not favor HFD after RYGB, according to our previous experience. In this dietary plan, body weight was increased by 15% in sham mice and decreased by 18% in RYGB mice at 6 wks post-surgery (Fig 3A). The weight change was a result of fat mass alteration (Fig 3B and 3C). There was no alteration in lean body mass (data not shown). Calorie intake was reduced in mice in the first week post-surgery (Fig 3D). RYGB mice exhibited a greater reduction in food intake and the reduction was significant at days 4 and 6 (Fig 3D). At 6 wks, calorie intake was identical between RYGB and sham mice. In addition, RYGB mice exhibited more preference to the chow diet (Fig 3D).

### Food-induced GIP mRNA in intestine

**Fig 4:**
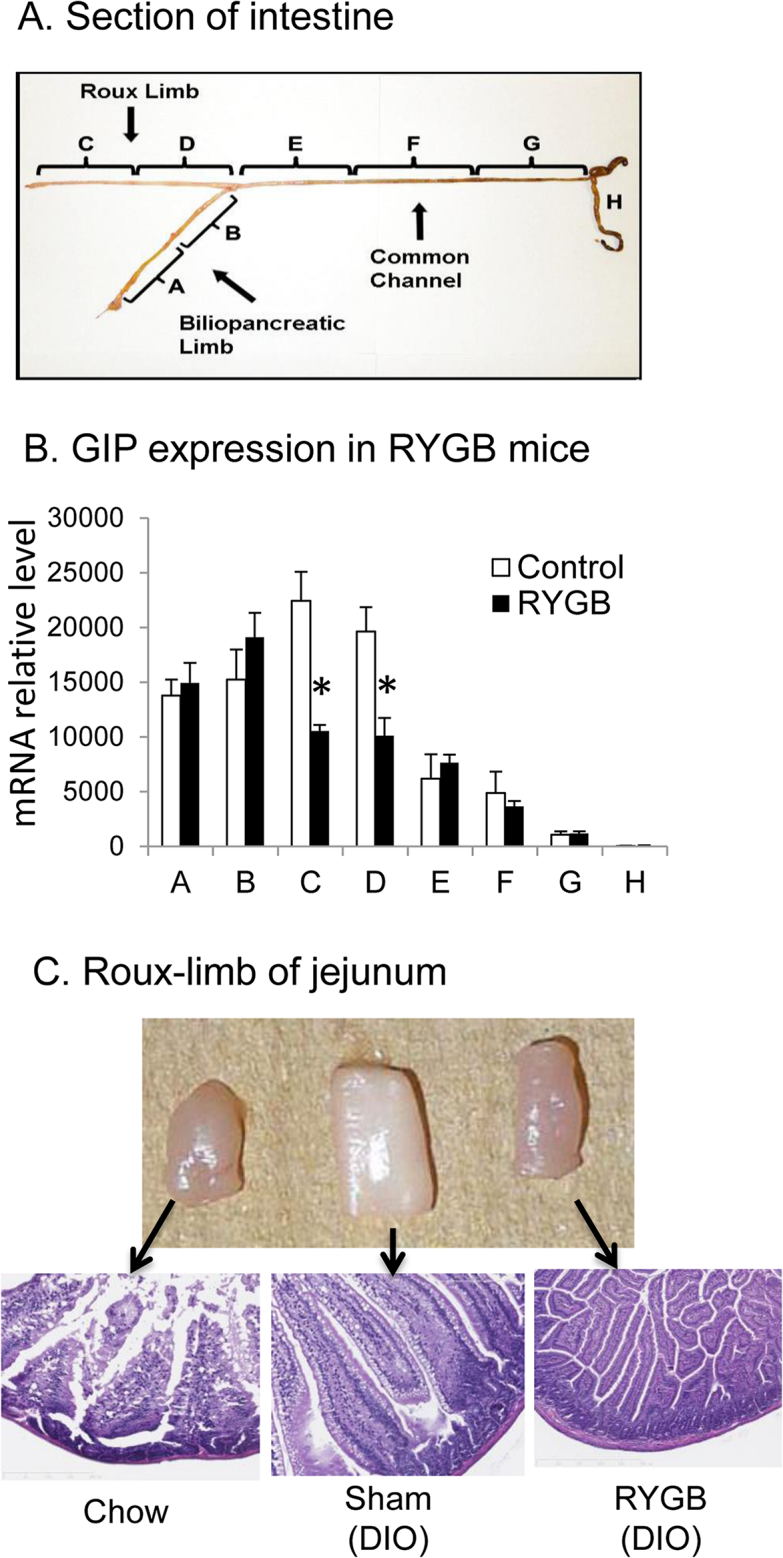
GIP mRNA in DIO mice after RYGB surgery (Cohort 2). GIP mRNA was measured in mice at 6 wks after surgery when the body weight was stabilized. The intestine samples were collected in the non-fasting condition. A. Intestine fragments are indicated in the diagram. B. GIP mRNA in different intestinal sections after RYGB surgery. Data is presented as mean ± SE (n = 5). *p<0.05 RYGB vs. sham by student’s t test. C. Histology of small intestine. The intestine fragments corresponding to Roux-limb are shown with microscope images (10X) of HE staining.

To study GIP expression in intestine, we divided the intestine into 8 segments labeled by letters A through E in cohort 2 (Fig 4A). RYGB and sham mice were compared in GIP mRNA at 6 wks after surgery in the fed condition. In RYGB mice, food-induced GIP expression was reduced by about 50% in the Roux-limb compared with that of corresponding jejunal segments in sham mice (segments C and D in Fig 4B). The GIP expression was identical between RYGB and sham mice in other segments (Fig 4B). The same results were observed in cohort 4 (data not shown). Jejunal morphology was examined to understand the histological basis of GIP reduction in Roux-limb (Fig 4C). In DIO mice, the jejunal segment exhibited an increase in diameter and a change in color from the pink to white. The mucosal villous surface was increased in the segment. After RYGB surgery, the changes in diameter and color were corrected in the jejunal fragment (the Roux-limb). In the Roux-limb of RYGB mice, the mucosa exhibited a hypertrophy, but villous surface was reduced. The surface was even less than that of chow-fed mice, suggesting a result of exposure to undigested food in the Roux-limb.

### Induction of GIP mRNA and protein expression in jejunum by nutrients

**Fig 5:**
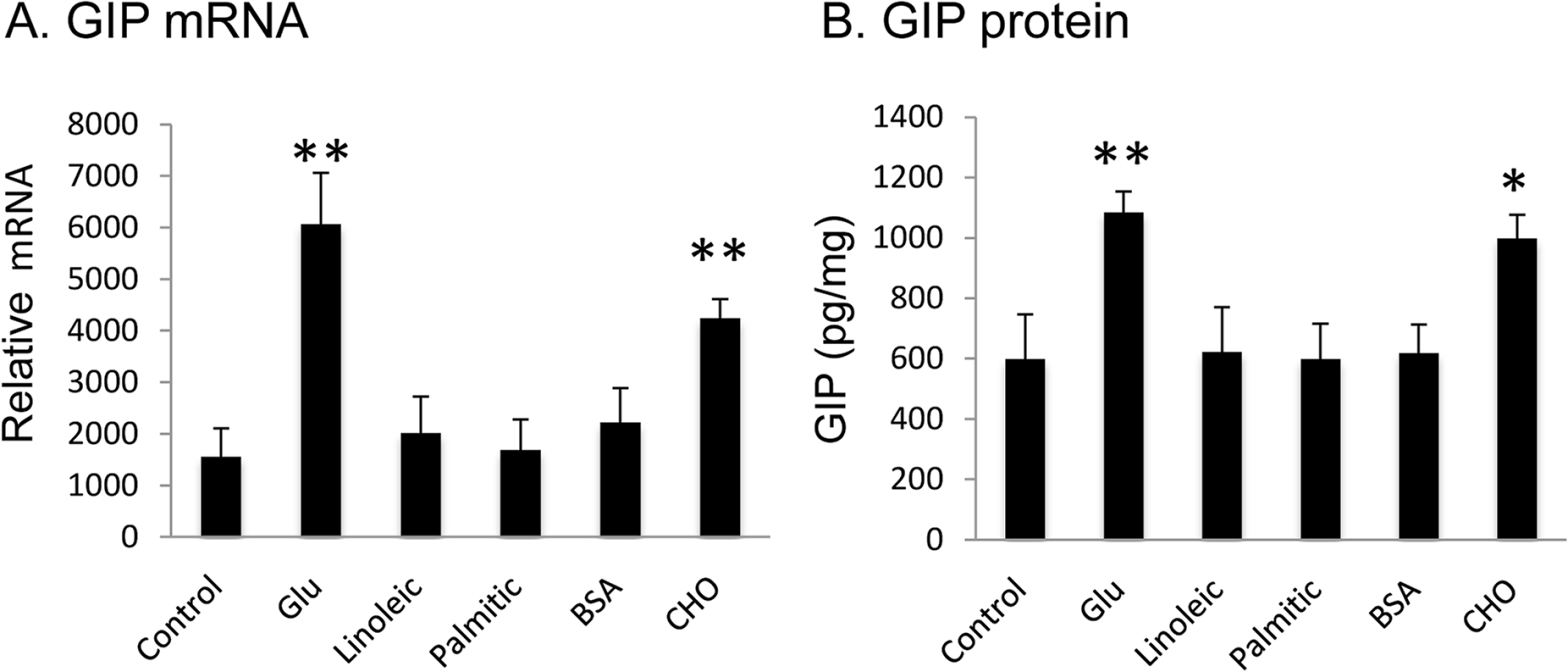
Induction of GIP expression by nutrients. GIP mRNA in tissue and protein in supernatant were examined in cultured jejunum tissues of chow-fed mice after nutrient treatment for 2 hrs. A. GIP mRNA in the tissue. B. GIP peptide in the culture supernatant. The concentrations of nutrients are glucose (Glu, 20mM), cholesterol (CHO, 5mM), linoleic acid (300μM), palmitic acid (100μM), and BSA (3%). Data is presented as mean ± SE (n = 6). * p<0.05; **p<0.001 vs. control by one-way ANOVA.

GIP expression is induced by food intake as suggested by increased GIP in the fed condition (Fig 1B). To understand the effect of nutrients on GIP expression, GIP mRNA was examined in jejunum in tissue culture. Glucose, free fatty acids (linoleic and palmitic acids), cholesterol, and BSA were used as nutrients to stimulate GIP expression. The mRNA was induced by glucose (3-fold, 20 mM) and cholesterol (2-fold, 5 mM), but not by linoleic acid (300 μM) and palmitic acid (100 μM) or BSA (3%) (Fig 5A). The mRNA expression was associated with an increase in GIP peptide secretion. GIP peptide was induced by glucose (1.8-fold) and cholesterol (1.6-fold), but not by the other nutrients (Fig 5B). The data suggests that glucose and cholesterol are effective stimuli to K cells in the induction of GIP expression.

### Postprandial GIP, PYY and ghrelin

**Fig 6:**
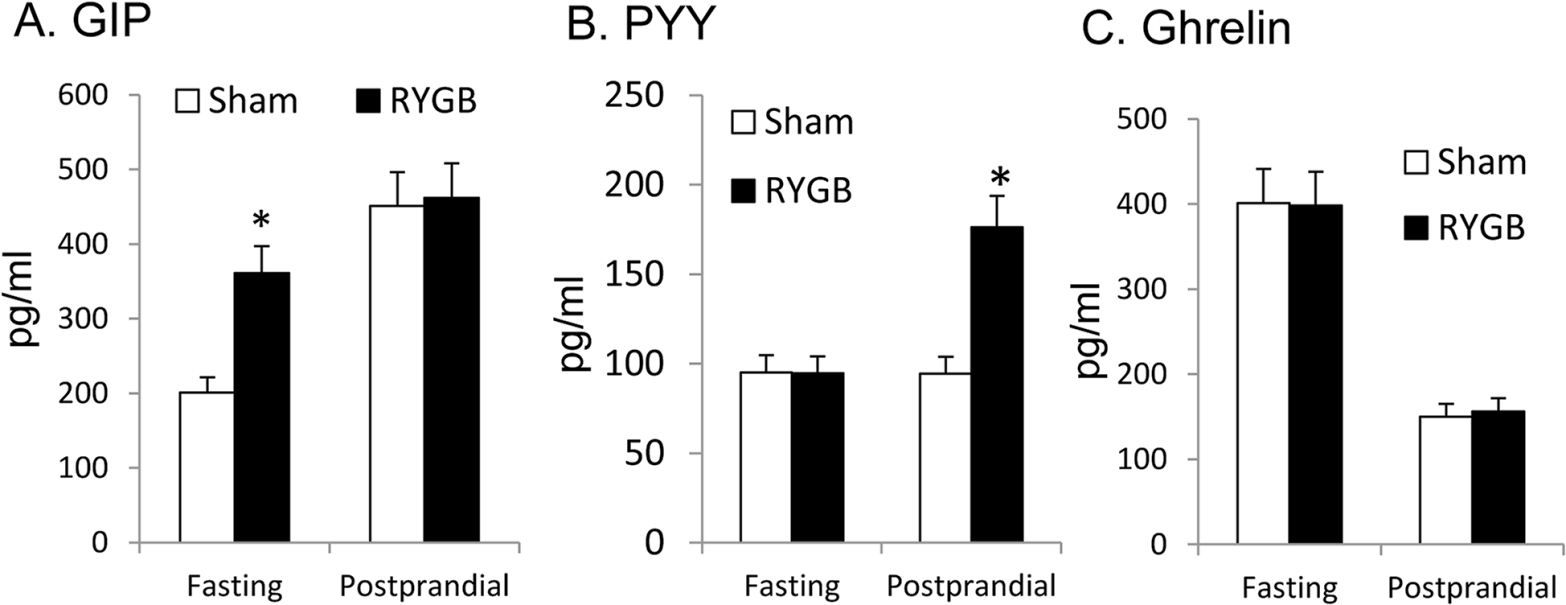
Serum GIP, PYY and ghrelin (Cohort 3). Serum GIP was tested together with PYY and ghrelin at 6 wks post-surgery. Serum was collected after overnight fast or at 30 mins of free feeding following overnight fast. A. Serum GIP. B. Serum PYY. C. Serum ghrelin. The result is presented as mean ± SE (n = 5–6). * p< 0.05 RYGB vs. sham by student’s test.

Gut hormones (GIP, PYY, Ghrelin and GLP-1) were determined in the fasting and fed conditions (30 mins chow meal) using serum samples collected at 6 wks post-surgery in cohort 3. RYGB mice exhibited a higher level of GIP in the fasting condition, but had identical GIP to sham mice in response to food intake (post-prandial) (Fig 6A). In cohort 4, serum GIP was tested at 15 mins after oral glucose gavage, and no difference was found between RYGB and sham mice (data not shown). RYGB mice and sham mice had identical serum PYY in the fasting condition, but RYGB mice exhibited 100% more PYY at 30 min upon feeding (Fig 6B). RYGB mice exhibited no difference to sham mice in serum ghrelin in both fasting and fed conditions (Fig 6C). The assay kit was not sensitive enough to detect serum GLP-1 in this study.

### Insulin sensitivity is improved by weight loss after RYGB

**Fig 7:**
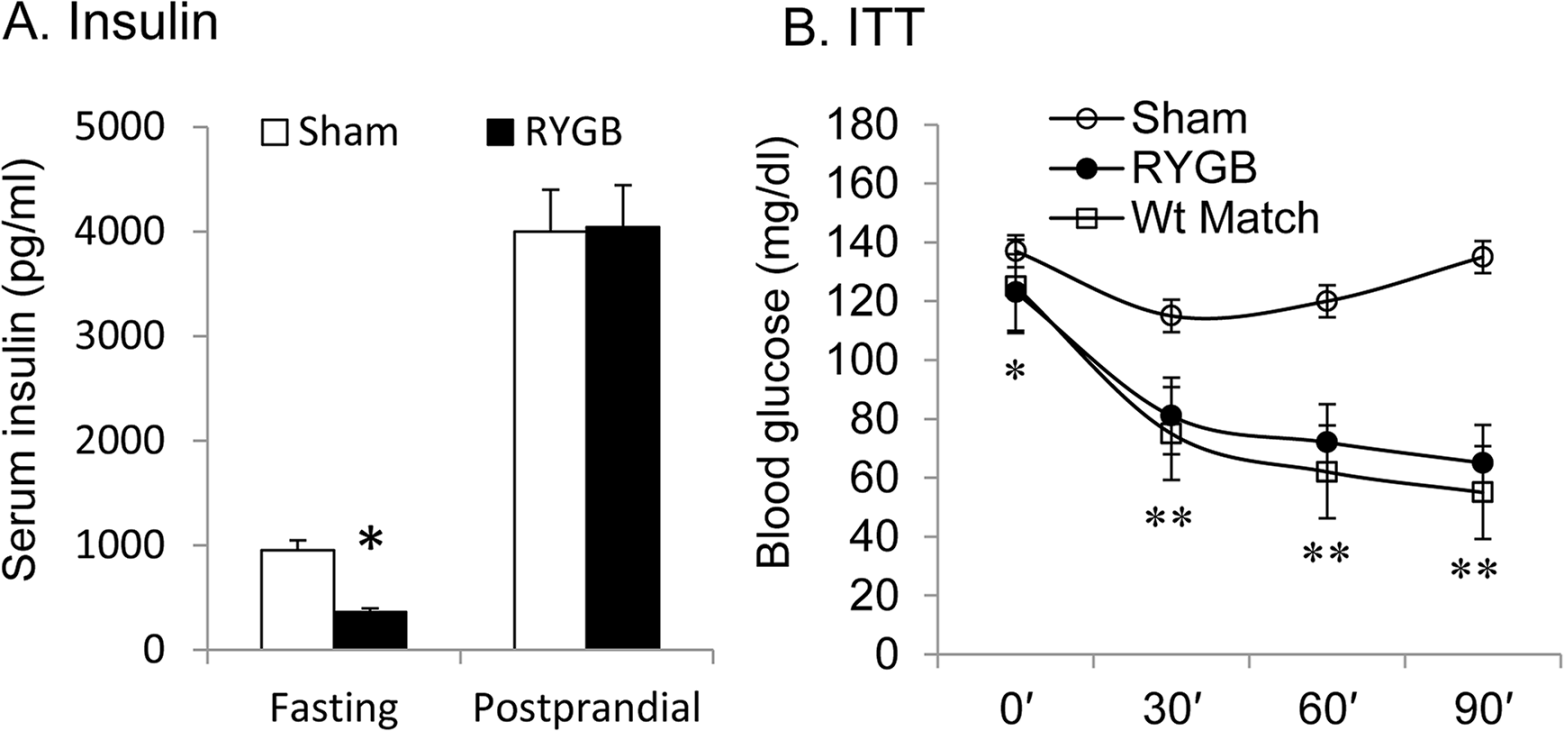
Insulin sensitivity (cohort 3). A. Fasting and postprandial insulin (n = 5–6). B. Insulin tolerance test. The test was conducted with i.p. insulin injection (0.8 U/kg) at 8 wks after RYGB surgery. Data is presented as mean ± SE (n = 7). * p<0.05; ** p<0.001 RYGB vs. sham by student’s test.

RYGB may improve insulin sensitivity with or without weight loss. In this study, we examined weight loss-dependent insulin sensitization by RYGB. Beside fasting insulin, insulin sensitivity was determined by insulin tolerance test at 6–8 wks after the surgery in cohort 3. Six weeks after surgery, when RYGB mice had lost about 30% of body weight, fasting insulin was reduced by more than 60% in RYGB mice, but postprandial insulin was not different between RYGB and sham animals (Fig 7A). Insulin tolerance was examined at 8 wks in mice of sham, RYGB and weight-matched groups. Weight-matched mice were generated in DIO mice through calorie restriction for an identical weight loss to RYGB mice. Insulin tolerance was significantly improved in both RYGB and the weight-matched mice over the sham mice (Fig 7B). Identical weight loss generated the same level of improvement in insulin tolerance in RYGB and the weight-matched groups.

## Discussion

In the present study, we examined GIP mRNA in the intestine to understand serum GIP response to RYGB surgery in a mouse RYGB model. We observed that GIP mRNA was elevated dramatically by HFD in the small intestine of DIO mice, which correlates to elevated serum GIP in obesity [17]. The expression was also induced by feeding. After RYGB, the feeding-induced GIP expression was reduced in the Roux-limb, but not in other segments of small intestine. The mRNA reduction was associated with the decrease in feeding-induced serum GIP response. Interestingly, serum GIP was higher in RYGB mice in the fasting condition with a higher basal level of serum GIP, which was identical to those of sham mice at 30 minutes during feeding or at 15 minute post oral glucose gavage. However, food intake failed to induce serum GIP in RYGB mice. Our data suggests that loss of stimulation by glucose and cholesterol in Roux-limb is likely responsible for the reduced serum GIP responses in RYGB mice. Alternatively, a change in non-intestinal GIP secretion cells may also play a role. Although the intestinal K cells are the major source of blood GIP proteins in mammalians [33], islet alpha-cells may be a potential source of blood GIP [33] as GIP mRNA and protein are found in islet alpha-cells in mouse and human [34]. Serum GIP is divided into active and inactive forms [35]. To better determine overall GIP secretion, total GIP protein was measured in current study.

Regarding the mechanism of GIP reduction in the Roux-limb of intestine in RYGB mice, lack of pancreatic enzymes and bile acids may be responsible the reduced availability of glucose and cholesterol in the stimulation of GIP expression. GIP expression and secretion are induced by nutrients [36]. We examined different nutrients (glucose, cholesterol, linoleic and palmitic acids) in the stimulation of GIP expression in intestinal tissue in tissue culture. The data suggests that glucose and cholesterol are active in the induction of GIP mRNA. The activity was not observed in linoleic and palmitic acids, suggesting that their activities in vivo [37] may be dependent on cholesterol in the bile acids [38]. Pancreatic enzymes and bile acids are not available in the Roux-limb due to surgery-induced anatomical changes in the small intestine. This prevents breakdown of dietary starch into glucose, and cholesterol release from dietary fat, which leads to reduced levels of glucose and cholesterol in the Roux-limb. The exposure to undigested food likely contributes to the mucosal hypertrophy and villous surface reduction in jejunum of Roux-limb in current study. The morphological change is consistent with that reported in RYGB patients by Spak, et al [39]. The nutritional and structural factors provide an explanation to the reduced GIP expression in the Roux-limb in RYGB mice. The elevated basal GIP in fasting condition may be a result of hypertrophy of Roux-limb mucosa to increase K cell numbers. Examination of K cells in Roux-limb will help to address this issue. Unfortunately, we were unable to identify a relevant antibody for such an assay. In a study of jejunal-ileal bypass (JIB), the bypass loop did not exhibit a difference from the control jejunum in rats [40].

This study suggests that GIP expression in the gut is gradually decreased along the intestine from duodenum to large intestine. It is known that GIP distribution in human gut follows this order: duodenum, jejunum, ilium and large intestine [33]. GIP distribution in dog, rat and mice are similar to that of human [33, 41, 42]. However, current study suggests that in mice, jejunum may express more GIP than duodenum, which is similar to that of pig.

The data could not exclude GIP activity in the maintenance of weight loss by RYGB. Gut hormones (GIP, GLP-1, PYY, etc) are actively studied in the mechanism of RYGB effects [11, 43]. In a recent study, we reported that GLP-1 is not required for RYGB-induced weight loss in pharmacological models and GLP-1R knockout mice [16]. In current study, RYGB and sham mice had identical serum GIP in postprandial condition although RYGB mice had a higher basal GIP in the fasting condition. The role of GIP remains to be tested in the long-term effect of RYGB. PYY was used as a positive control of RYGB-induced gut hormone. Our data confirms that serum PYY was increased in postprandial RYGB mice, which is consistent with findings in mice, rats and humans by other groups [8, 13, 27].

The data suggests that the negative energy balance is a powerful factor in the maintenance of sustained insulin sensitivity in RYGB mice. The improved insulin sensitivity was associated with sustained fat loss in our DIO mice, which is in agreement with observations by other groups [4, 5, 44]. The weight loss is a result of increased energy expenditure and fecal energy loss in the absence of food reduction in our RYGB model [45]. In this study, weight-matched mice were used to study the effect of weight loss on insulin sensitivity. Although the mechanisms of weight loss are different in RYGB mice and weight-matched mice, identical weight loss generated the same effects on insulin sensitivity in the two groups of mice. RYGB mice did not exhibit any advantage over the calorie-restricted mice in insulin sensitization. Similar same observations have been reported in patients [46, 47]. The weight-independent effect of RYGB surgery [48, 49] was not examined in current study, but the weight-dependent effect was clearly supported by our data.

In conclusion, the present study suggests that food-induced GIP expression was reduced in Roux-limb of RYGB mice. The mechanism is related to the lack of glucose and cholesterols in Roux-limb after re-routing of jejunum. The reduced mRNA is likely responsible for the decreased serum GIP protein in response to feeding in RYGB mice. Although the post-prandial GIP response was reduced, RYGB mice had an identical serum GIP to the sham mice in fed conditions due to the higher basal level, which is likely a result of hypertrophy of mucosa in Roux-limb. The role of GIP remains to be tested in the metabolic effects of RYGB. This study supports that PYY nay play a role in the long-term effect of RYGB [13]. The study enforces that the negative energy balance is required for the insulin sensitization by RYGB surgery.
